# Glucose Uptake Activities of Bis (2, 3-Dibromo-4, 5-Dihydroxybenzyl) Ether, a Novel Marine Natural Product from Red Alga *Odonthaliacorymbifera* with Protein Tyrosine Phosphatase 1B Inhibition, *In Vitro* and *In Vivo*

**DOI:** 10.1371/journal.pone.0147748

**Published:** 2016-01-25

**Authors:** Feng Xu, Fang Wang, Zhenhong Wang, Wenshan Lv, Wei Wang, Yangang Wang

**Affiliations:** 1 Research and Education Department, The Affiliated Hospital of Qingdao University, Qingdao, China; 2 Department of Endocrinology, The Affiliated Hospital of Qingdao University, Qingdao, China; 3 Department of Continued Education, The Affiliated Hospital of Qingdao University, Qingdao, China; 4 Department of Hematology, The Affiliated Hospital of Qingdao University, Qingdao, China; USDA-ARS, UNITED STATES

## Abstract

**Background and Aims:**

Protein tyrosine phosphatase 1B (PTP1B) is a novel therapeutic target for type-2 diabetes, which negatively regulates the insulin signaling transduction. Bis (2, 3-dibromo-4, 5-dihydroxybenzyl) ether (BDDE), a novel bromophenol isolated from the Red Alga, is a novel PTP1B inhibitor. But the anti-diabetic effects are not clear. In the present study, we evaluated the *in vitro* and *in vivo* antidiabetic effects of BDDE.

**Methods:**

The insulin-resistant HepG2 cells were used to evaluate the *in vitro* antidiabetic effects of BDDE. MTT assay was used to determine the safety concentrations in HepG2 cells. Glucose assay kit was used to check glucose uptake after treated with BDDE. Western blotting assay was used to explore the potent mechanisms. The db/db mice were used to evaluate the *in vivo* antidiabetic effects of BDDE. Body weight, blood glucose, Glycated hemoglobin (HbA1c), lipid profile, and insulin level were checked at the respective time points. Gastrocnemii were dissected and used to analyze the PTP1B and insulin receptor β (IRβ) expression.

**Results:**

BDDE increased the insulin-resisted glucose uptake in HepG2 cells. BDDE also decreased the expression of PTP1B and activated the substrates and downstream signals in insulin signal pathway, such as IRβ, insulin receptor substrate-1/2 (IRS1/2), phosphoinositide 3-kinase (PI3K), and protein kinase B (PKB/Akt). In the db/db mice model, BDDE significantly decreased the blood glucose, HbA1c and triglyceride (TG) levels. BDDE also decreased the expression of PTP1B and activated the phosphorylation of IRβ in gastrocnemii. Moreover, BDDE at high doses downregulated the body weight without affecting food and water intake.

**Conclusion:**

Our results suggest that BDDE as a new PTP1B inhibitor improves glucose metabolism by stimulating the insulin signaling and could be used in the treatment of type-2 diabetes mellitus.

## Introduction

Diabetes is a huge and growing problem all over the world, and the costs to society are high and escalating year by year [1]. Type-2 diabetes is the most common type of diabetes, which the body is able to produce insulin but either this is not sufficient or the body is unable to respond to its effects, leading to a build-up of glucose in the blood [2]. Thus, glycaemic control is the basis for the treatment of type-2 diabetes. Based on existing antidiabetic agents are often associated with side effects or insufficiency [3]. There is therefore a need for new drugs for type-2 diabetes prevention and treatment.

Protein tyrosine phosphatase 1B (PTP1B), a negative regulator of insulin signaling, has become the intense pharmaceutical interest for treating type-2 diabetes over the past decade [4, 5]. In insulin signaling pathway, both the insulin receptor β (IRβ) and insulin receptor substrate 1 (IRS-1) are substrates of PTP1B. The insulin pathway is activated when insulin binding to their receptor, following by auto-phosphorylation and activation. The activated insulin receptor promotes tyrosine phosphorylation of insulin receptor substrate-1 (IRS-1), leading to phosphatidyl-inositol 3 kinase (PI3K) and AKT activation as well as downstream lipid and glucose metabolism. In contrast, PTP1B dephosphorylates the IRβ and IRS-1, thereby attenuates the insulin signaling pathway [6].

Bis (2,3-dibromo-4,5-dihydroxybenzyl) ether (BDDE) is a novel bromophenol first isolated from the Red Alga *Odonthalia corymbifera* [7]. Kurihara et al and Kim et al investigated that BDDE is a potential α-glucosidase inhibitor [8–10]. Shi et al also found that BDDE displays PTP1B inhibition effects in an enzymatic activity assay [11]. Above all, these findings indicated that BDDE could be used in the treatment of type-2 diabetes mellitus. In the present work, we investigated the anti-diabetic properties of BDDE in insulin-resistant HepG2 cells and db/db mice as a PTP1B inhibitor.

## Materials and Methods

### Materials

BDDE was kindly provided by Dr. Fan, Institute of Oceanology, Chinese Academy of Sciences. HepG2 cells were purchased from BOSTER, Ltd. (Wuhan, China). Dulbecco’s modified Eagle’s medium (DMEM) and fetal bovine serum (FBS) were purchased from Invitrogen (Carlsbad, CA, USA). MTT, metformin and insulin were purchased from Sigma-Aldrich (St. Louis, MO, USA). The antibody directed against total IRβ, phospho-IRβ, total IRS-1, phospho-IRS1/2, total PI3K, phospho-PI3K, total Akt, and phospho-Akt (Ser473) was purchased from Cell Signaling Technology (Danvers, MA, USA). The antibody directed against GAPDH was purchased from Abcam Trading Company Ltd (Shanghai, China). A glucose assay kit was purchased from Solarbio (Beijing, China). All the other reagents were purchased from Sigma, unless otherwise indicated.

### Cell culture and Viability Assay

HepG2 cells were maintained in DMEM supplemented with 10% FBS, 100 units/mL penicillin, 100 μg/mL streptomycin at 37°C in a humidified atmosphere of 5% CO_2_ air. Cell viability was checked by the 3-(4,5-dimethylthiazol-2-yl)-2,5-diphenyltetrazoliumbromide (MTT) assay. Briefly, HepG2 cells were seeded onto 96-well plates at a density of 4 × 10^3^ cells/well for 12 h. Then the cells were treated with 1.25–100 μM of BDDE dissolved in DMSO (final concentration of DMSO: 0.1%). After 48 hours, the MTT was added and cell viability was calculated by dividing the absorbance of cells exposed to the test compound by the absorbance of control cells exposed to the DMSO alone.

### Induction of Insulin-Resistant HepG2 Cells

Induction of Insulin-Resistant HepG2 Cells was prepared as described previously with some modification [12]. Briefly, HepG2 cells were seeded onto six-well plates at 6 × 10^5^ cells/well or in 96-well plates at (2 × 10^4^ cells/well). After overnight incubation, the cells were then serum-starved for 24 h and pretreated for 24 h in serum-free DMEM with normal (5.5 mM) or high (30 mM) concentrations of D-glucose. Then the cells were cultured in 100 mg/L insulin for another 24 h, and harvested for assays as described below.

### Glucose Uptake Assay

The glucose uptake rate was measured using the glucose assay kit (glucose oxidase method) according to the published procedure with some modification [12]. Briefly, the insulin-resistant HepG2 cells were cultured in serum-free, high-glucose DMEM with or without BDDE at the specified concentrations or metformin (10 μM) for 24 h. After 24 h, the glucose concentration in the culture medium was measured using the glucose assay kit. The glucose uptake rate was calculated by subtracting the glucose concentration at the end of the culture period from the glucose concentration of the high-glucose DMEM. MTT assay was performed to adjust the errors generated from cell number difference.

### Western Blotting

Briefly, the insulin-resistant HepG2 cells were treated with or without BDDE and metformin. After 24 h, cells were lysed with ice-cold RIPA buffer (Solaibo, Beijing, China). Protein concentration was determined by using BCA Protein Assay Kit ((Thermo Scientific, Waltham, MA, USA). Cell lysates were separated by SDS-PAGE and transferred onto a NC membrane (Millipore, Billerica, MA, USA). The membranes were blocked with 5% skim milk in 1 × Tris-buffered saline containing 0.1% Tween 20 (TBST) for 1 h at room temperature and were then incubated overnight at 4°C with a primary antibody. The following day, the membranes were washed with TBST and were probed with a secondary antibody. The bands were detected using enhanced chemiluminescence reagents (Thermo Fisher Scientific Inc., Shanghai, China). Protein bands were visualized by autoradiography and the intensities were analyzed by Quantity One (Bio-Rad Laboratories, Hercules, CA, USA).

### *In Vivo* Experiment

All the experiments received prior approval from the Committee for the Humane Care and Use of Animals of Biological/Pharmacological Research Laboratories, The Affiliated Hospital of Qingdao University, in accordance with the Chinese Law on Humane Treatment and Management of Animals.

Db/db mice and their non-diabetic controls (db/dm mice) (6-week-old) were purchased from Experimental Animal Center of Military Medical Sciences (Beijing, China). At this age, db/db mice are already obese with elevated plasma insulin and glucose. Blood glucose was checked once a week to monitor the condition after induction of diabetes. In db/db mice models, the blood glucose levels were three times higher than their non-diabetic controls.

The db/db mice were kept in temperature-controlled cages (20–22°C) with a reversed light-dark cycle (8pm-8am) and with free access to water and food. The db/db mice were randomly divided into four groups, each group consisting of five mice. BDDE (10 and 40 mg/kg) and metformin (10 mg/kg) were administered orally to the mice once a day for 4 weeks.

Food intake, water intake and body weight were measured once a week. Blood samples were collected from the orbital venous plexus on Days 7, 14 and 28. Blood glucose was checked with ONE TOUCH Ultra^®^ glucometer (LifeScan, USA). Serum triglyceride (TG) and total cholesterol (TC) levels were measured by an enzymatic method (Whitman Biotech Co., Ltd., Nanjing, China) every two weeks. HbA1c concentration was measured by HbA1c reagent (Whitman Biotech Co., Ltd., Nanjing, China) on day 28. The plasma insulin levels were determined using an ELISA kit (ExCell Biology Inc., Shanghai, China) day 0 and 28.

Mice were anesthetized with isoflurane and euthanized by decapitation. Gastrocnemii were dissected, frozen in liquid nitrogen and stored at -80°C for later western blotting analysis. The gastrocnemii (30 mg) were homogenized in 300μl ice-cold RIPA lysis buffer supplemented with a protease inhibitor cocktail by using a tissue grinder placed on ice. Protein concentration was determined by using BCA Protein Assay Kit. Then, the western blotting assay was performed in accordance with the above introduction.

### Statistical Analysis

All the experiments were performed at least three times, and the data are presented as means ± SD values. Differences between the mean values were assessed using one-way analysis of variance. For all the analyses, p < 0.05 was considered significant. Statistical analyses were performed using SPSS 17.0 (SPSS, Inc., Chicago, IL, USA).

## Results

### Cytotoxicity of BDDE on HepG2 Cells

**Fig 1:**
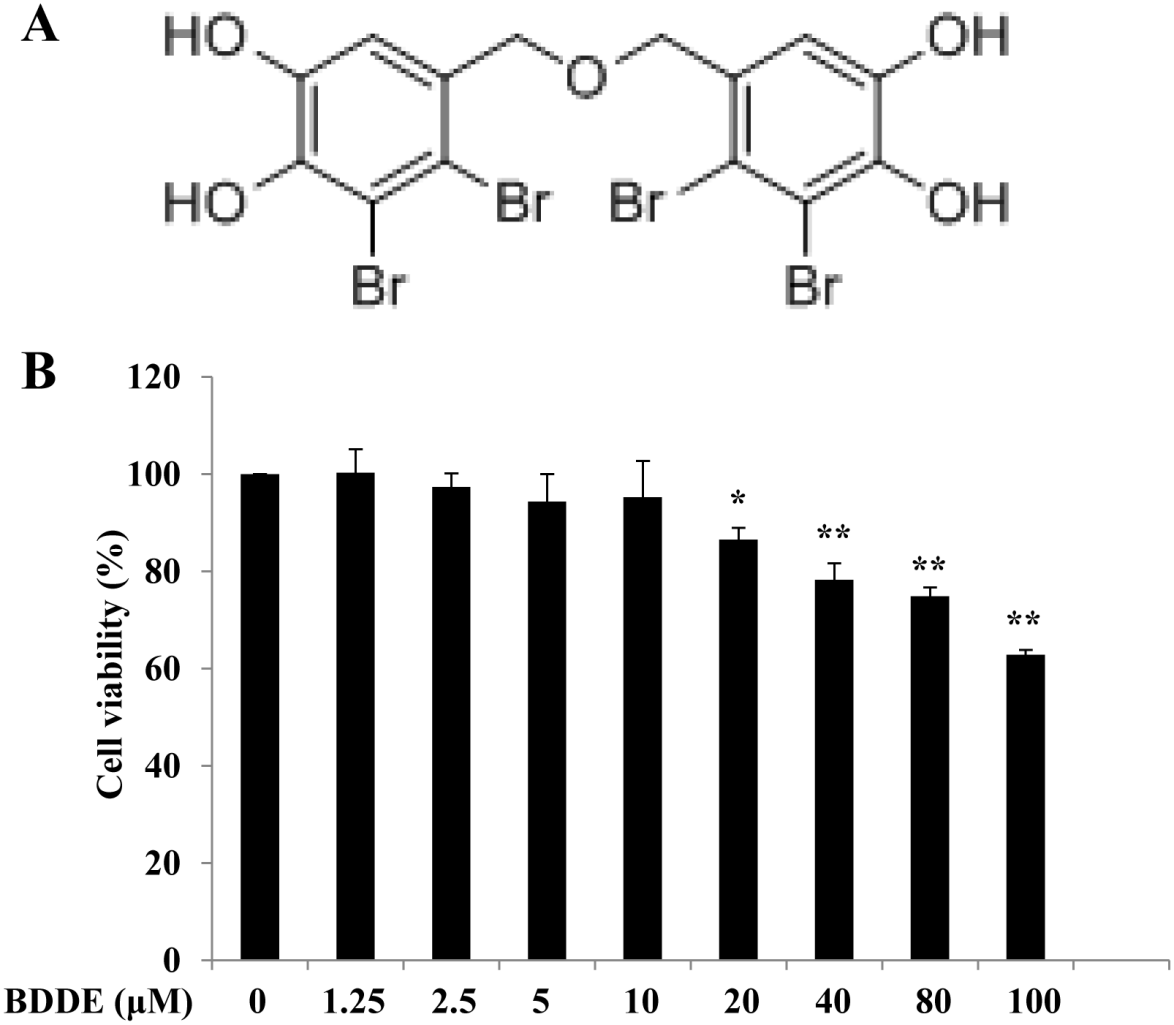
BDDPM did not reduce the survival of HepG2 cells up to 10 μM. (A) Structure of BDDE; (B) The HepG2 cells were treated with BDDE; after 48 h incubation, cell viability was evaluated with the MTT Assay. The data shown in the graphs are the mean ± SD values from at least three individual experiments. * p < 0.05, ** p < 0.01 versus the control.

To exploit the anti-diabetic effects of BDDE, we first examined the cytotoxicity of it on HepG2 cells. The HepG2 cells were treated with various concentrations of BDDE, and cell viability was measured by MTT assay. As shown in Fig 1, up to 10 μM treatment with BDDE for 48 h did not reduce the survival of HepG2 cells. Accordingly, further *in vitro* studies on the anti-diabetic activity of BDDE were conducted with 2.5, 5, 10 μM BDDE.

### BDDE enhanced glucose uptake in insulin-resistant HepG2 cells

**Fig 2:**
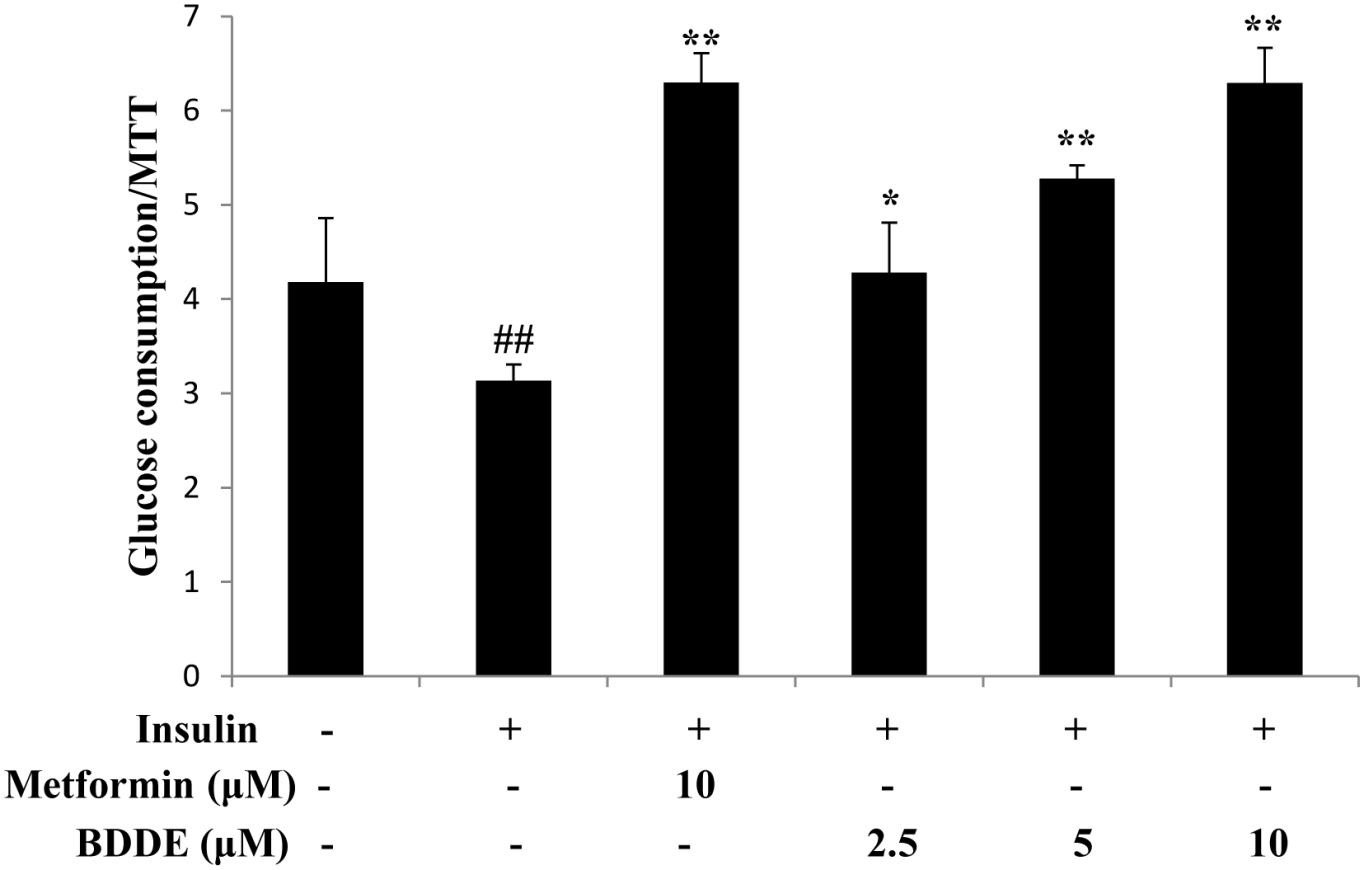
BDDE dose-dependently enhanced glucose uptake in insulin-resistant HepG2 cells. Data are presented as the mean ± SD of glucose uptake divided by the optical density determined in the MTT (n = 3). ## p < 0.01 versus control cells in the absence of insulin; * p < 0.05, ** p < 0.01 versus control cells in the presence of insulin.

We next examined the glucose uptake effects of BDDE in insulin-resistant HepG2 cells. The insulin-resistant HepG2 cells were built and treated with BDDE (2.5, 5, or 10 μM) and metformin (10 μM). After 24 h, BDDE significantly enhanced glucose uptake in a dose-dependent manner (Fig 2).

### BDDE inhibits PTP1B expression in insulin-resistant HepG2 cells

**Fig 3:**
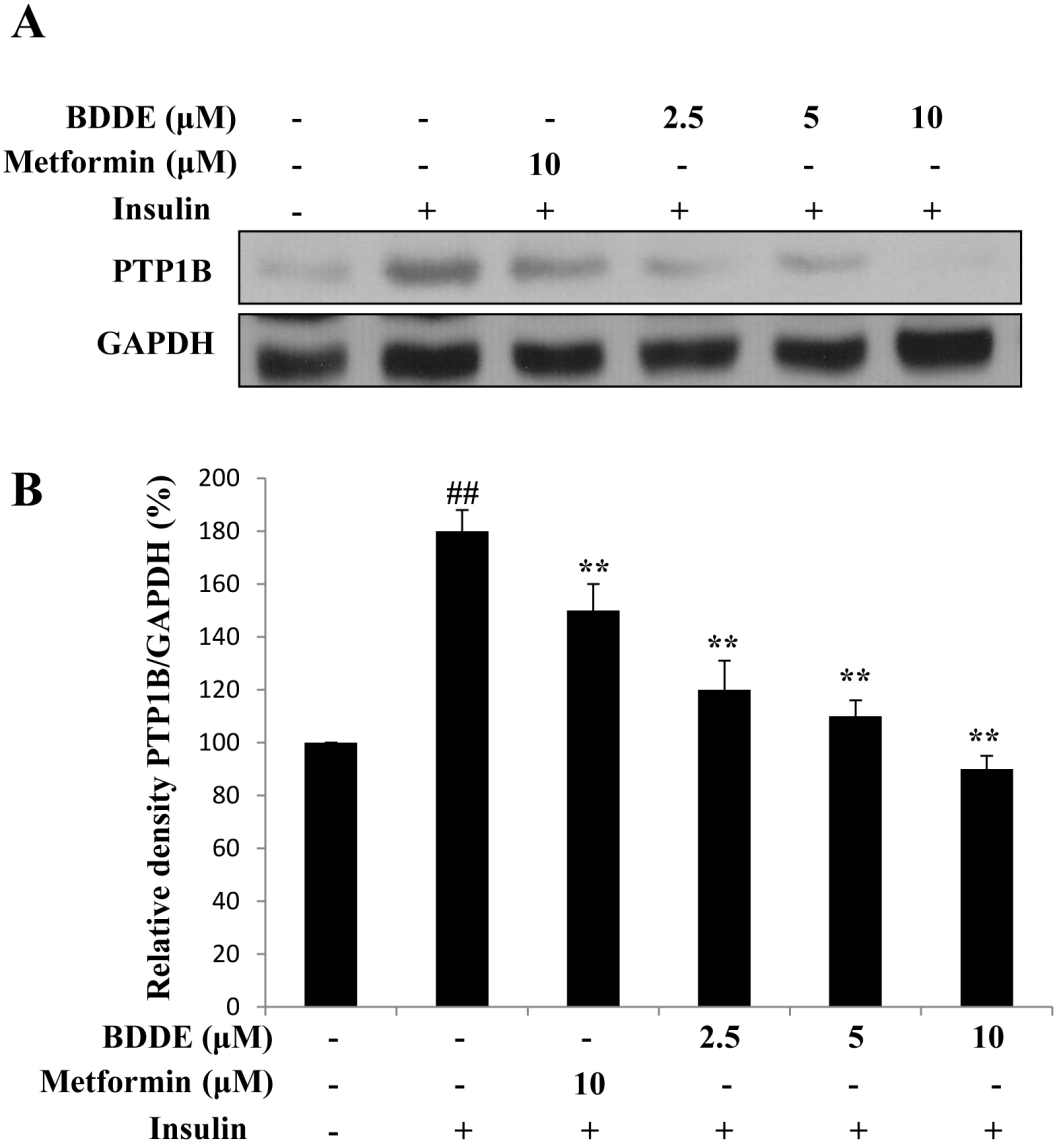
BDDE down regulated PTP1B expression in a dose-dependent manner. (A) The expressions of PTP1B are decreased after BDDE treatment; (B) The relative density of PTP1B to GAPDH. Data shown in the graphs are the mean ± SD values of at least three individual experiments. ## p < 0.01 versus control cells in the absence of insulin; ** p < 0.01 versus control cells in the presence of insulin.

PTP1B is an ideal therapeutic target for type-2 diabetes. It has been reported that BDDE can inhibit PTP1B enzyme activity. Here, we examined the PTP1B expression in insulin-resistant HepG2 cells. As shown in Fig 3, treatment with 2.5, 5, or 10 μM of BDDE for 16 h dose-dependently inhibited the over-expression of PTP1B in insulin-resistant HepG2 cells.

### BDDE down regulates the IRβ/IRS-1/PI3K/Akt signaling pathway

**Fig 4:**
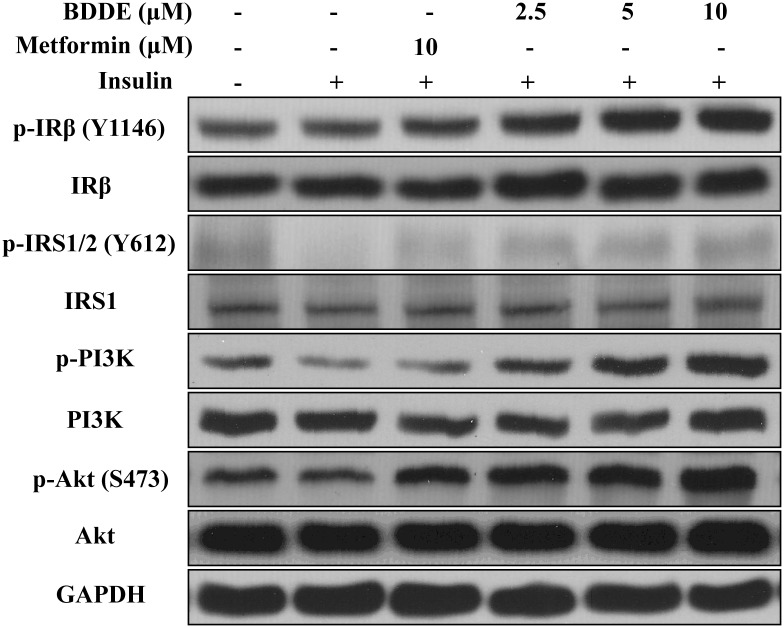
BDDE restored the phosphorylation of IRβ, IRS1, PI3K and Akt in a dose-dependent manner. HepG2 cells were treated with 0, 2.5, 5, 10 μM of BDDE for the indicated times. The levels of phosphorylated and total IRβ, IRS1, PI3K and Akt were determined by western blotting analysis.

IRβ and IRS-1 are two major substrates of PTP1B. We then explored whether the substrates and the downstream signals PI3K-Akt were affected by BDDE in insulin-resistant HepG2 cells. As shown in Fig 4 and S1 Fig, BDDE dose-dependently increased the expression of p-IRβ and p-IRS-1 in insulin-resistant HepG2 cells. BDDE also dose-dependently increased the expressions of p-PI3K and p-Akt, while did not affect the expression levels of total PI3K and total Akt.

### *In Vivo* Experiment of db/db Mice

**Fig 5:**
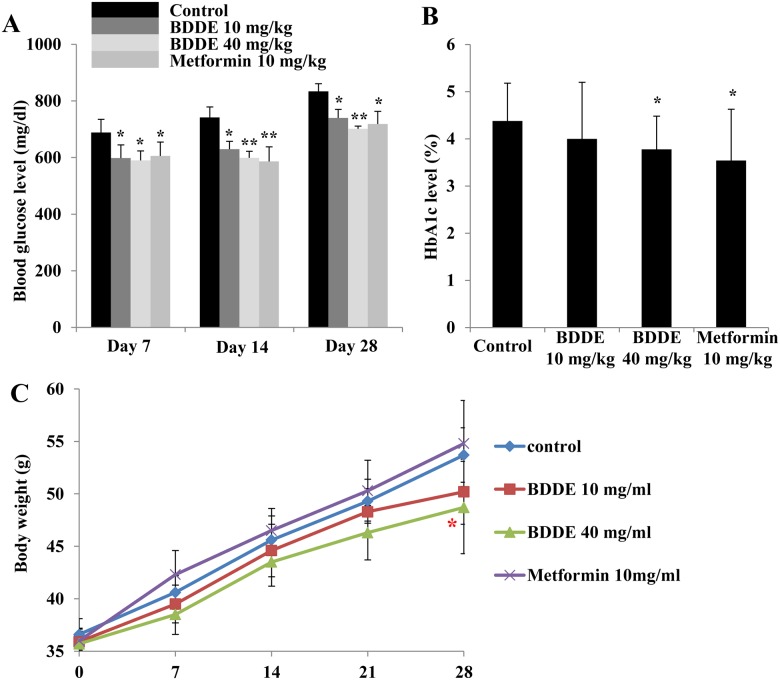
Effects of BDDE on blood glucose, HbA1c levels and body weight in db/db mice. BDDE was administered orally to db/db mice once a day for 4 weeks. The blood glucose (A), HbA1c levels (B), and body weights (C) were checked at the respective time points. Data shown in the graphs are the mean ± SD values of at least three individual experiments. * p < 0.05, ** p < 0.01 versus controls.

**Fig 6:**
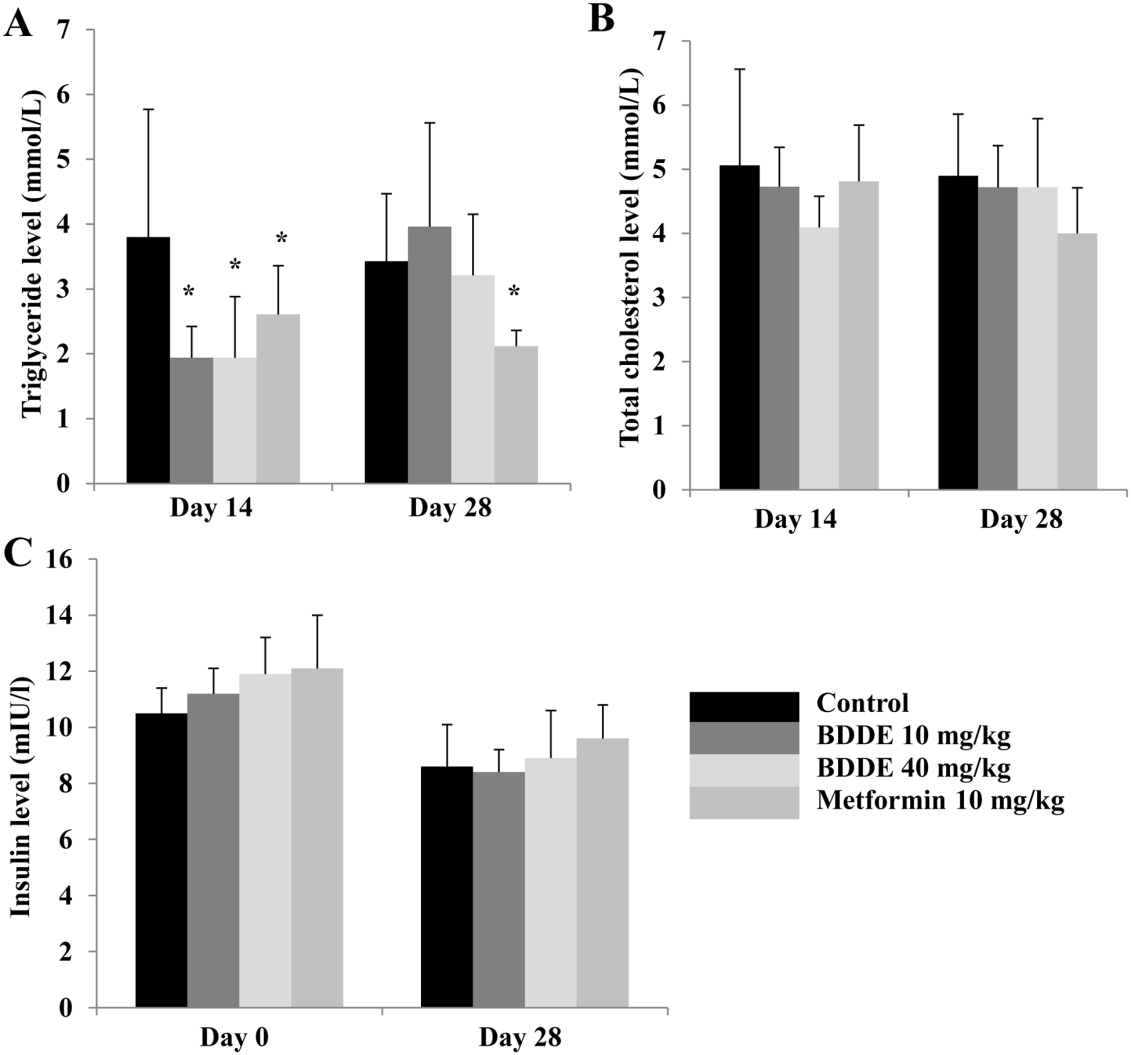
Effects of BDDE on triglyceride, total cholesterol and insulin level in db/db mice. BDDE was administered orally to db/db mice once a day for 4 weeks. The triglyceride (A), total cholesterol (B), and insulin levels (C) were checked at the respective time points. Data shown in the graphs are the mean ± SD values of at least three individual experiments. * p < 0.05 versus controls.

To further exploit the anti-diabetic effect of BDDE, we performed an animal experiment by using the db/db mice model. The blood glucose levels, HbA1c levels and the body weight at respective time points of the mice were checked. After treated with BDDE and metformin, a significant decrease in the blood glucose level was observed on Days 7, 14 and 28 (Fig 5A). On day 28, BDDE at high concentration were more effective in decreasing the blood glucose levels compared to metformin (702 mg/dl and 719 mg/dl, respectively). The HbA1c levels were decreased significantly in the metformin and BDDE 40 mg/kg-treated group, but not in the BDDE 10 mg/kg-treated group (Fig 5B). The body weight results were showed in Fig 5C. Although the body weights increased gradually during the treatment period, the mice in the metformin treated group had a higher body weight compared to the BDDE treated groups. Additionally, BDDE at different doses did not reduce the food and water intake in db/db mice, but metformin significantly decreased water intake on day 28 (S2 Fig). The results for TG, TC and insulin levels are show in Fig 6A, 6B and 6C. BDDE 10 mg/kg, 40 mg/kg and metformin treated groups significantly decreased the TG level on day 14. On day 28, only metformin treated group significantly decreased the TG level. Fig 6B and 6C showed that neither BDDE nor metformin could affect the TC and insulin levels during the dosing period.

**Fig 7:**
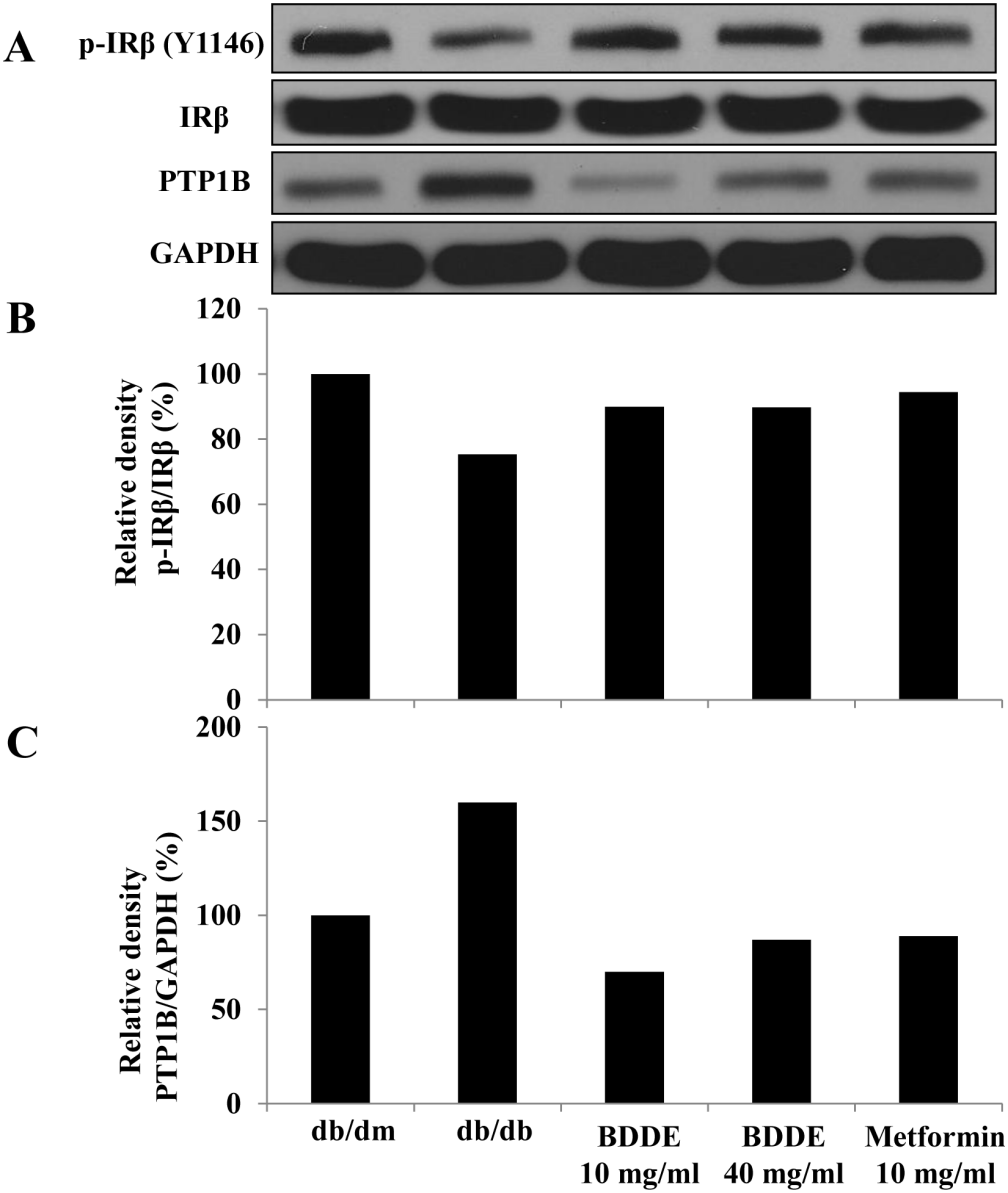
Enhancement effects of BDDE on the insulin receptor β (IRβ) phosphorylation and PTP1B expression in the muscle of db/db mice. The detected bands are shown in (A), The intensity was calculated as the ratio of the density of p-IRβ to the density of IRβ (B) and PTP1B to GAPDH (C).

A Western blotting assay was conducted to verify the expression of PTP1B and IRβ in muscle tissue to determine the inhibition effect of BDDE against PTP1B *in vivo*. As illustrated in Fig 7, p-IRβ expression in muscle tissue of db/db mice is lower than in that of db/dm mice, while PTP1B expression is much higher. P-IRβ expression was stimulated and PTP1B was decreased after treated with BDDE and metformin. These data suggested that BDDE inhibits PTP1B and activates the insulin receptor signal *in vivo*.

## Discussion

In this study, we investigated the anti-diabetic profiles of BDDE, which has been developed as a novel PTP1B inhibitor. First, BDDE enhances glucose uptake in insulin-resistant HepG2 cells. Second, BDDE inhibits PTP1B expression and down regulates the IRβ/IRS-1/PI3K/Akt Signaling Pathway *in vitro*. Third, BDDE exhibits anti-diabetic effects in db/db Mice, which significantly decreases the blood glucose levels, HbA1c levels, and TG levels. BDDE also inhibits PTP1B expression and activates p- IRβ expression *in vivo*.

Insulin resistance is the major inducement in causing type-2 diabetes. It is reported that PTP1B knockout mice remained insulin sensitivity [13]. In an animal models of type-2 diabetes, treatment with an antisense oligonucleotide specific for PTP1B results in improvement of hyper glycaemia and insulin sensitivity [14]. These results suggest that specific PTP1B inhibitors may thus be therapeutically beneficial in the treatment of type-2 diabetes. BDDE is a PTP1B inhibitor [11]. In order to explore the anti-diabetic effects, we first checked the glucose uptake in insulin-resistant HepG2 cells. BDDE increased the glucose uptake in a dose dependent manner. The glucose uptake concentration of BDDE was higher than the PTP1B inhibition value. These maybe because of the PTP1B is located in the endoplasmic reticulum (ER) surface, the cell membrane can block some compound. Considering that HepG2 cells was a cancer cell line, examination with other cell lines is necessary for further study.

It is well known that PTP1B dephosphorylates both phosphorylated IRβ subunit and phosphorylated IRS-1 to negatively regulate insulin signal transmission [15, 16]. In terms of profiling for a PTP1B inhibitor, it is critical that we evaluate whether the compound can enhance the auto phosphorylation of IRβ and its downstream signals, such as PI3K and AKT. In hepatic insulin resistance cells, the expression of PTP1B was markedly reduced after treated with BDDE. BDDE enhanced the phosphorylation of IRβ, IRS-1, PI3K and Akt. A western blotting assay in muscle tissue confirmed these results. These experiments revealed that BDDE inhibited the PTP1B and activated both the IRβ/IRS1 and PI3K/Akt signaling pathways in these cell line and db/db mice.

To further evaluate the anti-diabetic effect of BDDE, the compound was administered to db/db mice for 4 weeks. During the experimental period, BDDE showed good glucose and lipid control potential, which significantly improved glucose and TG metabolism and decreased the HbA1c levels without affection on body weight. Metformin also exhibited good glucose and lipid control effect, but increased the body weight compared to the BDDE treated groups, although the results were not significant. It is reported that PTP1B also involved in the leptin signaling, PTP1B-deficient mice are resistant to high-fat diet induced obesity [17]. Shi et al and S. Fukuda et al reported that the PTP1B inhibitors have body weight reduction potency in db/db mice [18, 19]. Our results showed that BDDE at high concentration reduced the body weight, which matched the previous studies. Our results also showed that the insulin level in db/db mice was not reduced. To fully understand these mechanisms, a further examination is necessary.

## Conclusions

In conclusion, we have elucidated the *in vitro* and *in vivo* anti-diabetic activities of BDDE. In insulin resistant HepG2 cells, BDDE significantly increased the glucose uptake by inhibiting PTP1B expression and activating the insulin signaling pathway. In diabetic mice, BDDE significantly reduced the blood glucose levels, HbA1c levels and TG levels without body weight enhancement. We expect BDDE to be useful in the treatment of type-2 diabetic. However, more researches are needed for this compound.

## Supporting Information

S1 Fig The ratio of phosphorylated IRβ to total IRβ, phosphorylated PI3K to total PI3K, phosphorylated IRS1/2 to total IRS1, and phosphorylated Akt to total Akt. (TIF)

S2 Fig Effects of BDDE on food and water intake. (TIF)
