# Supplementary figures and images for: Glucose Uptake Activities of Bis (2, 3-Dibromo-4, 5-Dihydroxybenzyl) Ether, a Novel Marine Natural Product from Red Alga Odonthaliacorymbifera with Protein Tyrosine Phosphatase 1B Inhibition, In Vitro and In Vivo
Source: PLoS One. 2016 Jan 25;11(1):e0147748. doi: 10.1371/journal.pone.0147748 (PMC4726511; doi:10.1371/journal.pone.0147748)

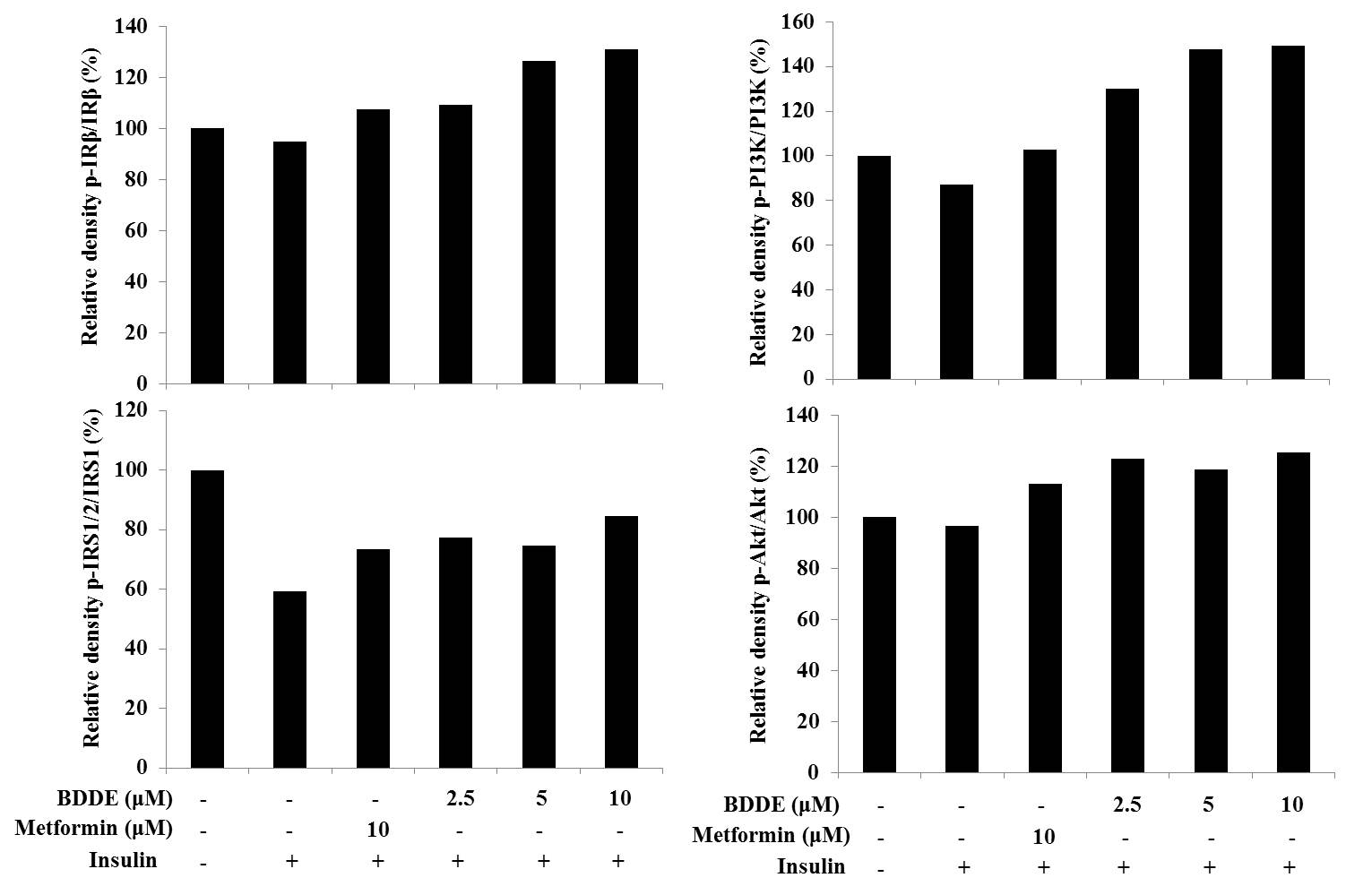

Supplement: S1 Fig — (TIF) [file pone.0147748.s001.tif]

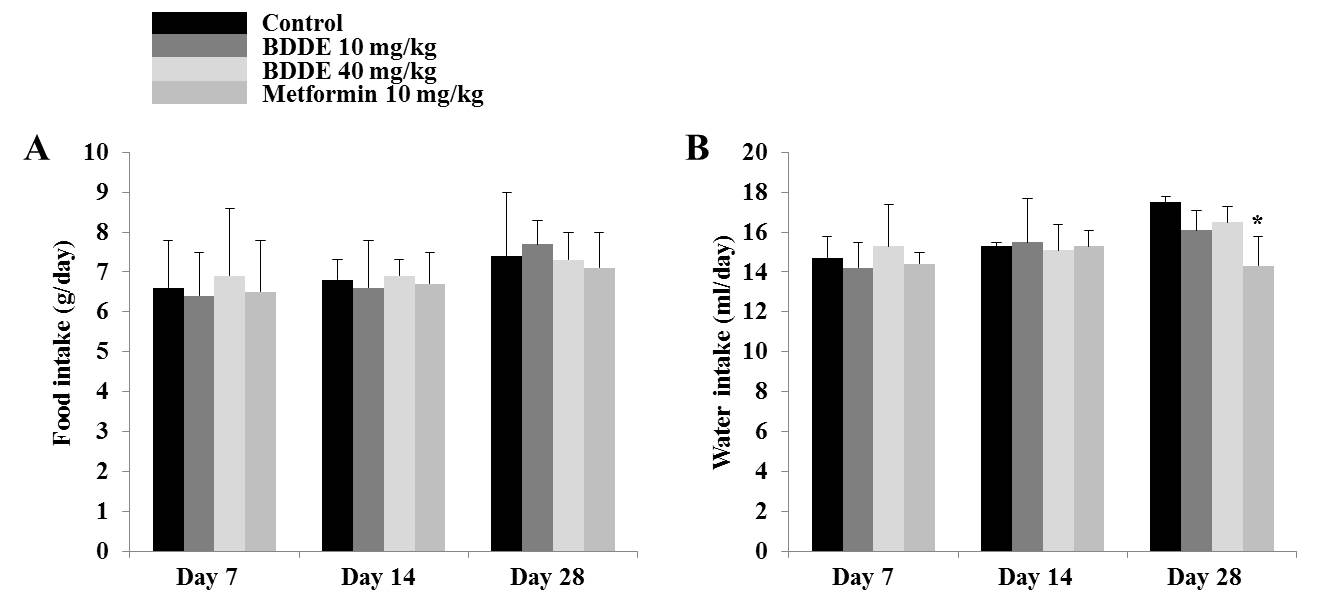

Supplement: S2 Fig — (TIF) [file pone.0147748.s002.tif]
